# Mindfulness-based stress reduction adapted to pregnant women with psychosocial vulnerabilities—a protocol for a randomized feasibility study in a Danish hospital-based outpatient setting

**DOI:** 10.1186/s40814-021-00860-w

**Published:** 2021-06-03

**Authors:** S. Skovbjerg, D. Birk, S. Bruggisser, A. L. A. Wolf, L. Fjorback

**Affiliations:** 1 The Danish Center for Mindfulness, Department of Clinical Medicine, Aarhus University, Aarhus, Denmark; 2 Department of Obstetrics and Gynecology, Copenhagen University Hospital, Hvidovre, Denmark

**Keywords:** Prenatal mental health, Pregnancy, Mindfulness, Mindfulness-based stress reduction, MBSR, Prenatal mindfulness-based stress reduction, Prenatal MBSR

## Abstract

**Background:**

This protocol is for a feasibility study of a mindfulness-based stress reduction (MBSR) program adapted for pregnant women with psychosocial vulnerabilities. The rationale for the study is the need for a wider array of evidence-based options to address prenatal mental health care needs in pregnant women. MBSR is a promising mental health intervention but has not yet been adapted for pregnant women with the aim of addressing prenatal mental health. The purpose is thus to evaluate the feasibility, acceptability, and clinical outcomes of an adapted MBSR program, prenatal MBSR, compared to usual care to inform a randomized controlled trial.

**Methods/design:**

Pregnant women (n = 60) referred to an outpatient clinic at Copenhagen University Hospital, Amager and Hvidovre, Denmark, will be recruited for the study. The design is a single-center feasibility trial, with prenatal MBSR, as an add-on to usual care. The primary outcome is to assess the feasibility of a full-scale randomized controlled trial. The secondary feasibility outcome includes possible effects of the adapted MBSR program estimated by self-report questionnaires measuring stress, anxiety, depression, well-being, decentering, reflective functioning, mindfulness, and compassion. Participants will be randomized in a 1:1 ratio to prenatal MBSR or usual care.

**Discussion:**

The study is part of the *Good Start to Family Life* study anchored at Copenhagen University Hospital, Amager and Hvidovre, Denmark. Teaching the skills of mindfulness meditation to a psychosocially vulnerable group of pregnant women could prove a viable and non-pharmacological approach to reduce stress, improve mental health, and provide support in the transition to parenthood. The outcomes of the feasibility study will inform the design of a fully powered randomized controlled trial.

**Trial registration:**

ClinicalTrials.gov, NCT04571190. Registered on September 30, 2020

**Supplementary Information:**

The online version contains supplementary material available at 10.1186/s40814-021-00860-w.

## Background

This protocol is for a feasibility study of an adapted mindfulness-based stress reduction (MBSR) program for psychosocially vulnerable pregnant women referred to a hospital-based outpatient clinic specialized in antenatal care. Reasons for referral to the outpatient clinic are mental disorders, substance abuse, or other psychosocial vulnerabilities such as domestic violence or a history of sexual abuse. The protocol is part of the *Good Start to Family Life* study, and the primary aim is to address the need of psychosocially vulnerable pregnant women and care providers. This in order to provide a wider array of evidence-based and non-pharmacological options to improve prenatal mental health. Mindfulness-based interventions are promising non-pharmacological interventions for mental disorders or symptoms, and performance has been found to be equivalent to evidence-based treatments, such as cognitive behavioral therapy and antidepressant medication for some disorders [1, 2]. Moreover, it is an acceptable intervention for pregnant women [3]. The adapted MBSR program will be referred to as prenatal MBSR.

Entering motherhood is one of the most intensive transitional stages in life that require adaptations on many levels [4], and this process is likely challenged by the simultaneous presence of psychosocial vulnerabilities. A history of any psychopathology or psychosocial adversities, including low social support and abuse, are predictors of mental disorders during and after pregnancy with little diagnostic specificity [5]. Stress and mental disorders in pregnancy have been associated with adverse outcomes such as preterm birth and/or low birth weight [6]. Moreover, it is an independent risk factor for later disruptions in child development including an increased risk for mental disorders [6, 7]. Mental disorders often have an early onset, compared to other chronic diseases, with about 50% starting before the age of 14 years and 75% before the age of 24 years [8]. From a societal perspective, early onset increases the years the individual is in need of support from health and welfare services. Consequently, reducing the risk of mental disorders is a crucial public health goal [8]. Evidence also  points to children experiencing multiple adversities in childhood having a markedly increased all-cause mortality risk in adulthood [9]. Outcomes from a meta-analysis on the effectiveness of interventions in mentally ill parents concluded that the risk of developing the same mental illness was reduced by 40% in the children [10]. This finding provides support for early intervention and prevention. The prenatal period is a sensitive period, and prevention of mental disorders during this time in psychosocially vulnerable pregnant women could possibly have a long-lasting impact for the benefit of both the individual, their families, and society [8].

Mindfulness can be conceptualized as an attentional skill [11], and the practice of mindfulness meditation is increasingly incorporated into mental health interventions [12]. MBSR was chosen as the “mother” program, because it is applicable to a broad range of conditions and contexts [12, 13]. It has a growing evidence base with meta-analyses consistently pointing to reductions in symptoms of stress, anxiety, and depression in both clinical and non-clinical populations [1, 14, 15]. MBSR was developed as an education and training vehicle for those suffering from chronic health problems and the mounting demands associated with psychological and emotional stress. The focus of MBSR is on learning how to relate to life challenges in new ways [16, 17]. One core feature of mindfulness meditation is the relationship between emotional suffering and the various strategies used to deal with it, e.g., distraction, repression, aggression, or somatic symptoms that may ultimately generate even more suffering [12]. One such maladaptive form of coping with emotional suffering is substance abuse. By training the capacity of becoming aware of one’s own mental state and developing a relationship with it can contribute to process suffering and ultimately improve mental health [11, 12]. In other words, the intention of mindfulness meditation training, in this context, is to engage and strengthen the pregnant women’s internal resources, thereby optimizing recovery or prevent relapse of mental health disorders during the perinatal period. Moreover, the intention of the training is also to teach skills that hold the potential to promote the formation of a healthy relationship with the child, i.e., present centered awareness and meta-awareness. When forming a deeper connection with oneself, a person is better able to connect with her/his child, and this quality of presence is at the heart of establishing a healthy bond.

More studies have addressed mental health in pregnancy using mindfulness-based interventions, e.g., MBSR; mindfulness-based cognitive therapy (MBCT); or mindfulness-based yoga. Systematic reviews and meta-analyses point towards a trend in favor of mindfulness-based interventions but state that shortcomings in study designs limit the conclusions. The majority of studies are pilot and feasibility trials that are not adequately powered to demonstrate the effectiveness [3, 18, 19]. A recent randomized controlled trial (RCT) examined the effect of a Mindfulness-Based Childbirth and Parenting Program (MBCP), which is adapted from MBSR [20], in pregnant women at risk of perinatal depression. The results of the study showed that MBCP significantly reduced perceived stress and depressive symptoms when compared to an active control group [20]. However, more research is needed to explore the possible benefits of mindfulness-based interventions on mental health in pregnancy, and in particular in psychosocially vulnerable groups [3, 19]. Moreover, none of the studies has included measures of mother-fetus relation such as prenatal reflective functioning. This measure may serve as an early indicator of mother-child interaction [21]. This is a relevant outcome to explore given the possible negative impact on the fetus and offspring of maternal mental disorders during pregnancy. Reflective functioning is the operationalization of mentalizing [22]. Mentalizing is a fundamental capacity in our social environment. It refers to the capacity to understand others and ourselves as motivated by intentional mental states, such as feelings, desires, wishes, goals, and attitudes [22]. It is not a unitary construct but covers both affective and cognitive components. Mindfulness is one such affective component involving the ability to attend to one’s own internal mental states [22]. Imbalance in mentalizing processes has been suggested as key to the understanding of psychopathology irrespective of diagnostic category [23]. In summary, more research on the effect of mindfulness-based interventions, on mental health, in the perinatal period is needed and in particular in psychosocially vulnerable groups.

### Study aim and objectives

The primary aim of the study is to determine the feasibility and acceptability of the prenatal MBSR program delivered to psychosocially vulnerable pregnant women in a hospital-based setting. The objectives are to examine the following: (1) acceptance of trial participation; (2) acceptance of allocated interventions; (3) risk of bias: loss to follow-up in the study arms, acceptance and compliance with the intervention, i.e., attending ≥ 5 sessions; and (4) the extent of missing data leading to missing outcomes. The secondary aim is to explore the potential efficacy of the prenatal MBSR program on self-reported measures of stress, well-being, anxiety, depression, reflective functioning, mindfulness, and self-compassion. The outcomes of the feasibility study will inform the design of a fully powered RCT.

## Methods/design

### Design

**Fig. 1:**
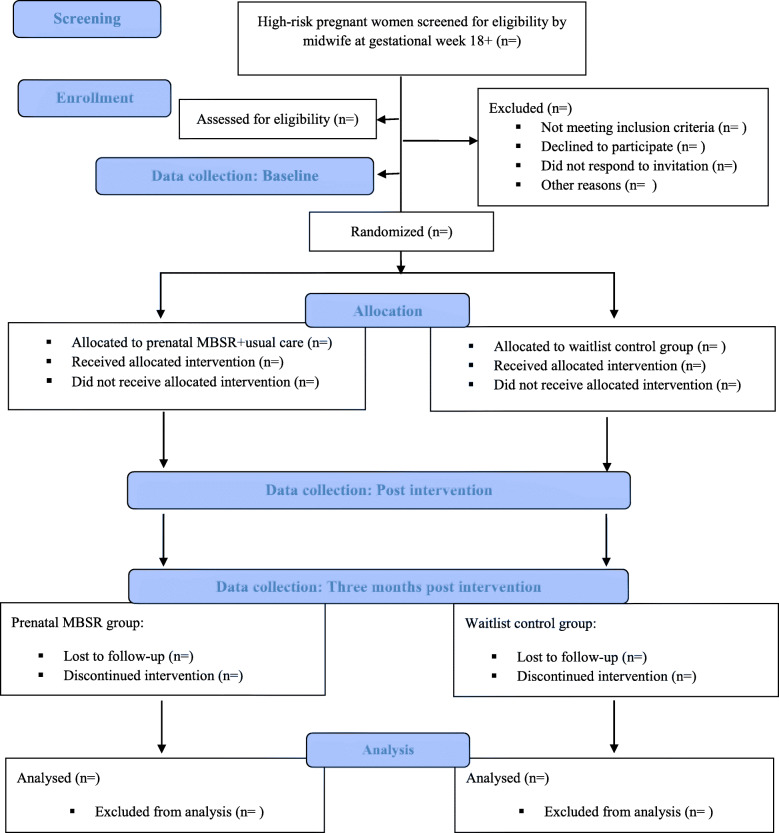
CONSORT 2010 flow diagram

The protocol is for a randomized, single-center feasibility trial comparing the prenatal MBSR program as an add-on to usual care with usual care alone. Participants will be randomized in a 1:1 ratio to an intervention or waitlist control group. A completed SPIRIT checklist is available in Additional Files and the study flow is outlined in Fig. 1. Self-report measures will be collected at three time points: (1) at baseline prior to randomization, (2) immediately after completion of the prenatal MBSR program, and (3) 3 months post-completion. Participants in the waitlist control group will be offered participation in a MBSR program after completion of the study.

### Setting

The study will be carried out at an outpatient clinic at Copenhagen University Hospital, Amager and Hvidovre, Denmark. The outpatient clinic is specialized in providing interdisciplinary antenatal care for psychosocially vulnerable pregnant women. In Denmark, there is free and equal access to most healthcare services including hospitals, general practitioners, and practicing specialists. The International Classification of Diseases, Tenth Revision (ICD-10) is used for the classification of diseases. The Copenhagen University Hospital, Amager and Hvidovre, is located in the greater Copenhagen area and is the country’s largest birthplace with 7000 annual births. Every year, about 800 pregnant women are referred to the outpatient clinic by their general practitioner with the first consultation being around 18 weeks’ gestation. Reasons for referral are past or present mental disorders, substance abuse, or other psychosocial vulnerabilities such as domestic violence or a history of sexual abuse. About 66% of all referrals are substantiated by a psychiatric diagnosis.

### Recruitment and consent

Pregnant women referred to the outpatient clinic are recruited at their first or second consultation with a midwife. The first consultation is on average approximately 18 weeks’ gestation and the second approximately 24 weeks’ gestation. In order to determine eligibility, the midwife will use the information from the patient record regarding reasons for a referral to the outpatient clinic. The information includes past or present psychiatric diagnoses, and past or present substance abuse. The information is registered in an Excel sheet along with name and contact information and passed on to the responsible researcher. The midwife will provide verbal information about the study and email written information to eligible women, who express an interest in participating in the study. An online zoom interview is then scheduled with the responsible researcher. The women will be encouraged to find a time and a place where the interview can take place undisturbed, and information is provided about the possibility of having a partner or other person present. The online format provides the women with an opportunity to participate in the interview in their own homes as they may feel particularly vulnerable during the corona pandemic. The corona pandemic, coupled with our experience working with this population, led to offering this option. If an online interview is not possible, the interview will be conducted at the outpatient clinic. The interview will contain information about the study such as study criteria, procedures, randomization, intervention, and motivation for study participation. Women who consent to study participation will be asked to sign the consent form within 1 week from the interview. The women can choose to either return a signed consent form by email or sign the consent form at the outpatient clinic, who will then return it to the responsible researcher.

### Inclusion and exclusion criteria

The aim of the study is to address prenatal mental care in psychosocially vulnerable women with no particular attention to any specific psychiatric diagnosis or other psychosocial vulnerability. However, there are some cases where MBSR is not suited, which is specified in the exclusion criteria.

The following are the inclusion criteria:

- Estimated due date no sooner than 3 months from the start of the intervention. The adapted MBSR program includes nine weekly sessions, and these criteria were included in order for the women to be able to complete the intervention.
- Eighteen + years of age.
- Speak and write Danish.
- Available for group intervention scheduled sessions. Being unavailable for two or more sessions is a reason for exclusion from study participation.
- Written informed consent to study criteria.

The following are the exclusion criteria:

- Concurrent substance abuse
- Psychotic disorders and post-traumatic stress disorder
- Suicidality

### Criteria for discontinuing allocated interventions

The criteria for discontinuation of study participation are (1) if requested by the participant, and (2) if the participant is absent from the prenatal MBSR class for two consecutive sessions and does not respond to email or phone calls.

### The intervention

#### The prenatal MBSR program

When adapting MBSR to high-risk pregnancies, the essential program elements characterizing MBSR were maintained [16] and integrated with the particular context and specific needs of pregnant women. The process of adapting MBSR is described in detail in the following.

The adaptations draw upon both empirical data as well as existing research and theory. The process of adapting the MBSR program was led by a psychiatrist, with extensive clinical and MBSR teaching experience, in collaboration with psychologists and researchers with relevant clinical and/or MBSR teaching experience.

In terms of empirical data, all staff at the outpatient clinic initially participated in a standard MBSR program. The purpose was for the staff to get a sense of what mindfulness training involves, by experiencing it for themselves, and, further, to generate knowledge that could inform the organization of an MBSR course for psychosocially vulnerable pregnant women. Thus, two focus group interviews with the staff were conducted after finalizing the 8-week MBSR course. The interview questions assessed the potential barriers for program attendance, the structure of the program, how to provide the best support to ensure attendance, and criteria for success.

The prenatal MBSR program has been pretested in five groups of pregnant women referred to the outpatient clinic. There was a maximum of 15 participants in each group. A psychiatrist and a psychologist with extensive clinical experience and MBSR teaching experience taught the groups. A midwife or a psychologist, from the outpatient clinic, participated in each of the groups. Pre- and post-self-report measures were collected. Attendance and dropout rates were systematically registered. Subsequent individual interviews with the participants and participating staff resulted in some adjustments to the program. In sum, the developmental process resulted in the following adaptations to the standard MBSR program: The first adaptation is that the prenatal MBSR program consists of nine 2-h sessions and no full-day  session. The rationale for including shorter sessions and no full-day sessions is to make the program more adaptable for the clinical context that it is intended for. The second adaption relates to the fact that transition to motherhood is often presented as an exciting and joyful time with naturally developing emotional growth by modern society and the media [24]. The idealistic image may not be a close representation of the actual experience for women with mental health or severe psychosocial problems. Therefore, the program emphasizes a particular focus on the mother-child relationship by drawing attention to the bodily sensations, emotions, and thoughts associated with pregnancy and motherhood as they unfold. The third adaptation includes a yoga program that is suitable for pregnancy. , The fourth includes shortened sitting meditations  and greater alternation, during sessions, between meditation exercises in order to accommodate the bodily changes associated with pregnancy. The fifth adaptation includes a reduced 15-min daily mindfulness practice, with options for longer practice; this is to make it more manageable considering the vulnerabilities and life circumstances of these women. Lastly, the sixth adaptation to the program is that it is delivered in a combination of physical attendance (sessions 1, 4, and 9) and online delivery. Combining physical attendance with online delivery reduces traveling time and ensures attendance. This is while still providing a good starting point for establishing a learning environment and the feeling of being part of a group. Online booster sessions will be offered every 3 months after finalizing the 9-week program and continues throughout the first postnatal year. Mothers are invited to bring their babies to the booster sessions thereby making it an opportunity to practice being mindful with their babies.

#### Instructor qualifications

The prenatal MBSR program will be delivered by two highly trained MBSR teachers from the Danish Center for Mindfulness, Aarhus University. Both have been involved in the process of adapting the MBSR program to the present context and have extensive clinical psychiatric experience.

#### Usual care (TAU)

Standard clinical practice, usual care (TAU), consists of routine pregnancy visits to the outpatient antenatal clinic at Copenhagen University Hospital, Amager and Hvidovre, and to a general practitioner. A multidisciplinary approach involves primarily preventive counseling by midwives and, in some cases, consultations with a physician or social worker throughout the pregnancy and follow-up until the early post-partum period. In some cases, usual care may include consultations with a psychologist, although usually limited to a few sessions, and in more severe cases, referral to psychiatric treatment. All consultations are individual, and the first session is planned in gestational week 18, with an average of six following consultations. Additional consultations are planned if deemed necessary.

### Primary outcome: feasibility of a full-scale RCT

The primary outcome of the study is to access the feasibility of a full-scale RCT, including accessing acceptance of trial participation among psychosocially vulnerable pregnant women. Acceptance of trial participation includes recruitment rate, acceptance of allocated interventions, compliance (i.e., attending ≥ 5 sessions), non-completion, and adverse events.

The recruitment process will be systematically registered in close collaboration between the Danish Center for Mindfulness, Aarhus University, and the outpatient clinic at Copenhagen University Hospital. Project meetings, every other week, will provide an opportunity for continuous evaluation of the recruitment process.

Prenatal MBSR class attendance will be registered at each session, and participants will be asked to notify the teacher in case of non-participation. Participants canceling two consecutive sessions will be contacted by phone, and the responsible midwife will be informed in order to provide support and prevent dropout. Adherence and acceptability of the prenatal MBSR program will be evaluated after the trial is finalized by examining attendance, time spent on home practice, and overall satisfaction with the program. On a daily basis, participants will be asked to register time spent on home practice. Satisfaction with the program will be evaluated at the last session. The prenatal MBSR teacher will register any adverse event ascribed to the prenatal MBSR program and notify the responsible researcher.

### Secondary feasibility outcome

The secondary feasibility outcome includes the possible effects of the prenatal MBSR program as estimated by self-report measures assessing stress, anxiety, depression, well-being, decentering, reflective functioning, mindfulness, and compassion. The questionnaire packet is built and managed via REDCap (Research Electronic Data Capture) hosted by Aarhus University. Reminders will be emailed after 1 week in case of non-response.

#### The Perceived Stress Scale

The Perceived Stress Scale 10-item version (PSS-10) is a global measure of stress [25]. The questions concern thoughts and emotions experienced within the past month such as “In the last month, how often have you felt that you were unable to control the important things in your life?” The response format ranges from “never” (= 0) to “very often” (= 4). Scores range from 0 to 40 with higher scores pointing to more perceived stress. The Danish consensus version of the PSS-10 can be used in clinical research settings and has good psychometric properties [26].

#### The Edinburgh Depression Scale

The Edinburgh Depression Scale (EDS) is a widely used screening questionnaire containing questions on how the respondent has felt in the past 7 days [27]. The questionnaire has 10 items scored 0–3 on each item yielding a maximum score of 30. A higher score points to more depressive symptoms, and a score of 11 has been suggested as the optimal cutoff for depression according to both DSM-5 and ICD-10 criteria [28]. The EDS has been validated as a screening instrument for prenatal and postpartum depression, showing good psychometric properties [29].

#### Depression Anxiety Stress Scales

The Depression Anxiety Stress Scales (DASS-21) [30] has three subscales designed to discriminate between depression, anxiety, and stress in the last week. The DASS excludes somatic items such as sleep disturbance, lack of energy, and poor concentration, which may not be valid markers in pregnancy or the postpartum period [31]. Each subscale of depression, anxiety, and stress includes seven items. Response to each item is rated on a 4-point Likert scale ranging from “never” to “very much/most of the time.” Scores are calculated for each subscale, and higher scores point to more symptoms of stress, anxiety, or depression. The DASS-21 has shown good psychometric properties when administered to young adults in non-clinical settings [32].

#### The World Health Organization – Five Well-Being Index (WHO-5)

The WHO-5 is a short and generic global rating scale measuring subjective well-being [33]. It consists of five statements, and the respondent is asked to rate how well each of the statements applies to him or her when considering the last 14 days. Each item is scored from 5 “all of the time” to 0 “none of the time.” Final scores range from 0 to 100 with higher scores representing greater well-being. The scale has adequate validity both as a screening tool for depression and as an outcome measure in clinical trials and has been applied successfully across a wide range of study fields [33].

#### Prenatal Reflective Functioning Questionnaire

Parental reflective functioning refers to the caregiver’s capacity to reflect upon his/her own internal mental experiences as well as those of the child [34]. The Prenatal Parental Reflective Functioning Questionnaire (P-PRFQ) is a recently developed, 14-item questionnaire for assessing early parental mentalizing capacity [4]. Responses are rated on a scale from 7 “strongly agree” to 1 “strongly disagree.” The raw scores range from 14 to 98 and the index sum from 1 to 7. Higher scores indicate higher prenatal mentalizing capacity. The P-PRFQ has shown good initial psychometric properties [4].

#### The Experiences Questionnaire: subscale for decentering

Decentering reflects the capacity to take a non-judgmental and accepting stance regarding one’s thoughts and emotions as opposed to identifying with them [35]. The decentering subscale, of the experiences questionnaire, consists of 11 items with responses ranging from 1 “strongly disagree” to 5 “strongly agree.” The total score ranges from 11 to 55 with higher scores indicating higher decentering. Initial support has been reported for the reliability and validity of the scale as a measure of decentering [35].

#### The Five Facet Mindfulness Questionnaire

The Five Facet Mindfulness Questionnaire (FFMQ) assesses five general facets of being mindful in daily life: observing, describing, acting with awareness, non-reactivity to inner experience, and non-judging of inner experience. Items are rated on a 5-point Likert scale ranging from 1 “never or very rarely true” to 5 “very often or always true.” Higher scores suggest higher levels of mindfulness. Previous studies have provided good support for the construct validity of the FFMQ; furthermore, four of the five facets (except for “acting with awareness”) have been found to be significantly correlated with meditation experience [36].

#### The Self-Compassion Scale

The Self-Compassion Scale measures the ability to have a healthy stand towards oneself that does not involve evaluations of self-worth [37]. The scale consists of 12 items, and responses are given on a 5-point scale from 1 “almost never” to 5 “almost always.” Higher scores indicate more self-compassionate behavior. The psychometric properties of the scale have been extensively evaluated [37].

#### Baseline characteristics

Baseline data on age, marital status, number of children, social support, working status, education, obstetric complications (e.g., gestational diabetes), current use of psychotropic medication, and prior and current history of a mental disorder or drug abuse will be collected. Assessment of health practices will include self-reports of level and type of habitual exercise, smoking, and alcohol. Information on time of birth (weeks) and birth weight will be collected postnatally.

### Sample size

The study will include a total of 60 participants. This sample size is justified based on the design and objectives of the study [38].

### Randomization

Eligible participants will be randomized in a 1:1 ratio to either prenatal MBSR + usual care or usual care alone using the REDCap randomization module hosted by Aarhus University. REDCap is a secure, web-based application designed to support data capture for research studies [39]. A data manager from Aarhus University, not otherwise involved in the study, programs the randomization algorithm. Inclusion and thus randomization will take place consecutively until the estimated number of participants is reached.

### Data analyses

Statistical analyses will be performed using SPSS for Windows (version 27; IBM Corp, Armonk, NY). Skewness, kurtosis, and visual inspection of normal Q-Q plots will be used to inspect normality. Descriptive statistics will be presented as mean and SDs or median and interquartile range (IQR) depending on the distribution of the continuous variables or as frequencies for categorical variables.

Recruitment, participation, and retention rates will be reported and presented in a CONSORT flow diagram [40]. Data collected on numbers of eligible participants recruited, numbers randomized, adherence to, and compliance with the intervention, as well as dropouts lost to follow-up, will be presented as percentages and used to inform the development of a full-scale RCT.

Acceptability of the intervention will be assessed by dropout rates, by computing the mean number of completed sessions, and time spent on at-home practices in the prenatal MBSR group. Participation in ≥ 5 sessions will be considered compliant with the program. A loss to 12-week follow-up analysis will be conducted for age, education, employment, history of a mental disorder, participation in < 5 sessions of the intervention, and pre-scores on the PSS-10 by means of t-test and chi-squared test (χ^2^). A loss to intervention analysis will be conducted for participants attending < 5 sessions of the intervention applying a similar approach.

Analyses of a possible effect of the intervention will be performed based on the intention-to-treat principle, meaning that all participants will be included in the analysis irrespective of their compliance with the intervention protocol. Lastly, an additional per-protocol analysis will be conducted. Since the study is not powered by formal hypothesis testing, only effect estimates and 95% CIs will be presented in accordance with the CONSORT criteria for reporting of feasibility or pilot studies [40].

Independent t-tests (or the non-parametric equivalent Mann-Whitney U) will be conducted to compare the pre- and post-questionnaire scores of the intervention and control groups. In case of skewed distributions in baseline variables, e.g., age and history of mental disorders, logistic regression analyses will be performed.

### Blinding

Group affiliation will be concealed for the researcher performing the statistical analyses.

Participants will not be blinded. Keeping participants blind to this type of intervention would not be possible. Part of the study is to understand if this introduces any difficulties (i.e., resentful demoralization) into the study that would need to be addressed before a larger trial.

### Scientific ethical considerations

Participation in a mindfulness-based program has not been associated with major side effects when the program is delivered by a certified teacher. The Danish Center for Mindfulness, Aarhus University, is affiliated with the Center for Mindfulness at Brown University and collaborates on setting international standards for education and teacher training. In the present study, a psychiatrist and a psychologist will be teaching the prenatal MBSR program. Both have extensive clinical experience and are certified mindfulness teachers. Some side effects have been reported in connection with mindfulness training, such as aggravation of symptoms that could be due to the increased focus on internal states and feelings of losing oneself. If any adverse events should occur, the teachers have the relevant expertise to ensure participant safety or referral to relevant treatment elsewhere. Both teachers are available for individual sessions between the program sessions. Any side effects related to the intervention will be systematically registered by the responsible researcher and published with the results of the study. The possible benefits of mindfulness training in pregnancy as described in earlier sections thus outweighs the potential side effects of the intervention. Moreover, there is a need for more evidence-based, non-pharmacological options for addressing prenatal mental healthcare needs in vulnerable groups for the benefit of both mother and fetus. The adaptations made to the original MBSR program require more research in order to establish an evidence base for the prenatal MBSR program, which is initiated with this feasibility study.

## Discussion

The evidence to date suggest that MBSR and mindfulness-based interventions have great potential in improving mental health in both clinical and non-clinical populations. There is a need for evidence-based and non-pharmacological options addressing mental health during pregnancy. Teaching the skills of mindfulness meditation to a vulnerable group of pregnant women could prove as a viable and non-pharmacological approach, to improve mental health. Moreover, it may provide support in the transition to parenthood. It is anticipated that the findings from the study will access and determine the acceptability and feasibility of the prenatal MBSR program and inform the design of a fully powered randomized controlled trial. The results could also be relevant for other hospitals or healthcare settings working with pregnancy both in and outside of Denmark.

### Status of the study

The study was initiated in mid-January 2021.

### Roles and responsibilities

The Danish Center for Mindfulness, Aarhus University, is responsible for the development and evaluation of the prenatal MBSR program. The developmental and evaluation process is carried out in close collaboration with the outpatient clinic at Copenhagen University Hospital, Amager and Hvidovre. The collaboration includes project meetings every other week and regular meetings with staff at the clinic. Project meetings are held with the responsible parties involved in the *Good Start to Family Life* Project including the management at the outpatient clinic, Oestifterne, and the Danish Center for Mindfulness. The involvement of the staff during the development of the prenatal MBSR program is described in the “The intervention” section. The Danish Center for Mindfulness will have full access to the final dataset.

## Supplementary Information

**Additional file 1.** SPIRIT Checklist.

## Data Availability

Not applicable
